# New Saccharin Salt of Chlordiazepoxide: Structural and Physicochemical Examination

**DOI:** 10.3390/ijms231912050

**Published:** 2022-10-10

**Authors:** Anna Lech, Patrycja Garbacz, Artur Sikorski, Maria Gazda, Marek Wesolowski

**Affiliations:** 1 Department of Analytical Chemistry, Faculty of Pharmacy, Medical University of Gdansk, Gen. J. Hallera 107, 80-416 Gdansk, Poland; 2 Department of Physical Chemistry, Faculty of Chemistry, University of Gdansk, Wita Stwosza 63, 80-308 Gdansk, Poland; 3 Institute of Nanotechnology and Materials Engineering, Faculty of Applied Physics and Mathematics, Gdansk University of Technology, Narutowicza 11/12, 80-233 Gdansk, Poland

**Keywords:** chlordiazepoxide, saccharin, organic salt, Powder X-ray Diffraction (PXRD), Single Crystal X-ray Diffraction (SCXRD), Differential Scanning Calorimetry (DSC), Thermogravimetric Analysis (TGA), Fourier Transform Infrared (FT-IR), Raman spectroscopy

## Abstract

Since the formation of organic salts can improve the solubility, bioavailability, and stability of active pharmaceutical ingredients, the aim of this work was to prepare an organic salt of chlordiazepoxide with saccharin. To achieve this goal, the saccharin salt of chlordiazepoxide was obtained from a physical mixture of both components by grinding them with a small volume of solvent and by crystallizing them with complete evaporation of the solvent. The resulting salt was examined by methods such as Powder X-ray Diffraction (PXRD), Single Crystal X-ray Diffraction (SCXRD), Differential Scanning Calorimetry (DSC), Thermogravimetric Analysis (TGA), Fourier Transform Infrared (FT-IR), and Raman spectroscopy. The results of the studies proved that saccharin salt of chlordiazepoxide crystallizes in the orthorhombic *P*bca space group with one chlordiazepoxide cation and one saccharin anion in the asymmetric unit. In the crystal of the title compound, the chlordiazepoxide cation and the saccharin anion interact through strong N–H···O hydrogen bonds and weak C–H···O hydrogen bonds. The disappearance of the N–H band in the FT-IR spectrum of saccharin may indicate a shift of this proton towards chlordiazepoxide, while the disappearance of the aromatic bond band in the chlordiazepoxide ring in the Raman spectrum may suggest the formation of intermolecular hydrogen bonds between chlordiazepoxide molecules. The melting point of the salts differs from that of the starting compounds. Thermal decomposition of the salt begins above 200 °C and shows at least two overlapping stages of mass loss. In summary, the results of the research showed that the crystalline salt of the saccharin and chlordiazepoxide can be obtained by various methods: grinding with the addition of acetonitrile and crystallization from acetonitrile or a mixture of methanol with methylene chloride.

## 1. Introduction

The low water solubility of active pharmaceutical ingredients is one of the most important challenges in solid dosage form technology [1]. Poor solubility results in low bioavailability of the drug substance as only a small fraction of the drug dose penetrates into the blood circulation. Overall, about 40% of the drug substances available on the pharmaceutical market are poorly soluble in water, while 70% to 90% of drug candidate compounds are also poorly soluble in water [2]. Many strategies have been proposed to improve the water solubility of poorly or practically insoluble drug substances. One promising strategy is the formation of organic salt. In this case, the physical, chemical, and biopharmaceutical properties of the medicinal substances are modified. Moreover, crystal salts differ from other crystal forms (polymorphs, co-crystals) in that the components in the ionized state (cations and anions) and with a specific stoichiometry form salts [3]. Generally, the solubility and stability of organic salts increase most frequently, as compared with medicinal substances [4].

**Figure 1:**
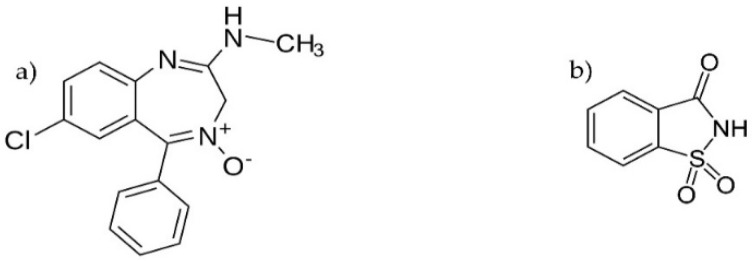
Formula of (**a**) chlordiazepoxide and (**b**) saccharin.

An example of a drug substance practically insoluble in water is chlordiazepoxide (IUPAC name: 7-chloro-2-methylamino-5-phenyl-3H-1,4-benzodiazepine-4-oxide, Figure 1a) [5]. It is a synthetic compound belonging to the group of benzodiazepines. Similarly to other benzodiazepines, chlordiazepoxide has a sedative, anxiolytic, anticonvulsant, and muscle relaxant effect in the human body [6]. Hence, benzodiazepines are used for anxiety disorders, insomnia, alcohol withdrawal, and seizures. Chlordiazepoxide is particularly useful in the treatment of alcohol withdrawal and anxiety disorders [7]. Moreover, in combination with mebeverine hydrochloride, it is used in the treatment of irritable bowel syndrome as a drug reducing tension and anxiety in the digestive system [8]. However, the too-widespread use of chlordiazepoxide in the past, especially in the treatment of anxiety disorders, has meant that today chlordiazepoxide is not the first-line drug in the treatment of anxiety disorders, but is a second-line drug [9]. Despite this, chlordiazepoxide is still a valuable drug substance for the treatment of anxiety disorders and the relief of alcohol withdrawal symptoms.

Taking into account the key therapeutic effect of chlordiazepoxide and the limitations of its use resulting from its low water solubility, the aim of the research was to improve the bioavailability of chlordiazepoxide by modifying its physicochemical properties. Therefore, it was decided to obtain the salts of chlordiazepoxide and saccharin by two methods for preparing organic salts, i.e., by grinding with a small volume of solvent and by crystallization with complete evaporation of the solvent.

Beneficial properties as a salt-forming compound has saccharin [10,11]. Similarly to chlordiazepoxide, saccharin is also a synthetic compound, 2-sulfobenzoic acid imide (IUPAC name: 1,1-dioxo-1,2-benzothiazol-3-one, Figure 1b). A valuable property of saccharin as a weak acid compound (*pK_a_* = 2.2) is the ability to transfer N–H hydrogen to the base, creating the saccharin anion. Regardless of this property, saccharin functional groups are capable of acting as hydrogen donors (N–H) and hydrogen acceptors (S=O, C=O), forming strong hydrogen bonds. In turn, chlordiazepoxide, as a weak base compound (*pK_a_* = 4.8 [12]), has functional groups capable of forming hydrogen bonds with saccharin, acting as a hydrogen donor (N–H, C–H) and a hydrogen acceptor (N^+^–O^−^) [13]. The choice of saccharin as an organic component of salts results from the fact that saccharin is readily soluble in water and is non-toxic with no negative effects on the human body [14]. It is worth noting that saccharin is the oldest chemical sugar substitute from the first generation of artificial sweeteners, at the same time the most widely used and about 450 times sweeter than sucrose [15]. Saccharin is a pharmaceutically acceptable compound; it is used as an ingredient of drug products and dietary supplements.

From the literature review, it appears that no organic salts of chlordiazepoxide have been obtained so far. Of the remaining benzodiazepines, the salts of olanzapine with organic acids [16] and alprazolam with dihydroxybenzoic acid [17] have been described. A significant increase in the solubility of these salts was indicated with compared to the solubility of the drug substance. On the other hand, there are numerous salts of saccharin with active pharmaceutical ingredients, such as quinine, haloperidol, mirtazapine, pseudoephedrine, lamivudine, risperidone, sertraline, venlafaxine, zolpidem and amlodipine [18], sulfamethazine [19], stanozolol [20], lamotrigine [21], trospium [22], diphenhydramine [23], ciprofloxacin [24], and palmatine [25].

## 2. Results and Discussion

The aim of the work was to obtain and examine a new saccharin salt of chlordiazepoxide, prepared in two ways—grinding with the addition of a small amount of solvent and crystallization with complete evaporation of the solvent. The crystal structure and thermal behavior of the newly obtained salt, the starting components (chlordiazepoxide and saccharin), and the physical mixture were examined by PXRD, SCXRD, DSC, and TGA methods. Additionally, spectroscopic methods such as FT-IR and Raman were used to study the formation of hydrogen bonds between saccharin and chlordiazepoxide. The results of diffractometric, thermoanalytical, and spectroscopic measurements confirmed the formation of salts between saccharin and chlordiazepoxide.

### 2.1. X-ray Diffractometric Measurements

Chlordiazepoxide and saccharin were analyzed by PXRD. Then, in the same way, the physical mixture obtained by 15 min mixing at 20 rpm using a laboratory stirrer and saccharin salts of chlordiazepoxide prepared by various methods were analyzed. PXRD patterns confirmed that both the chlordiazepoxide and saccharin used in this study were in the crystalline form. The compatibility of the diffraction pattern of the physical mixture with the diffraction patterns of the starting components confirmed that only crystalline forms of benzodiazepine and saccharin could be found in the mixture.

**Figure 2:**
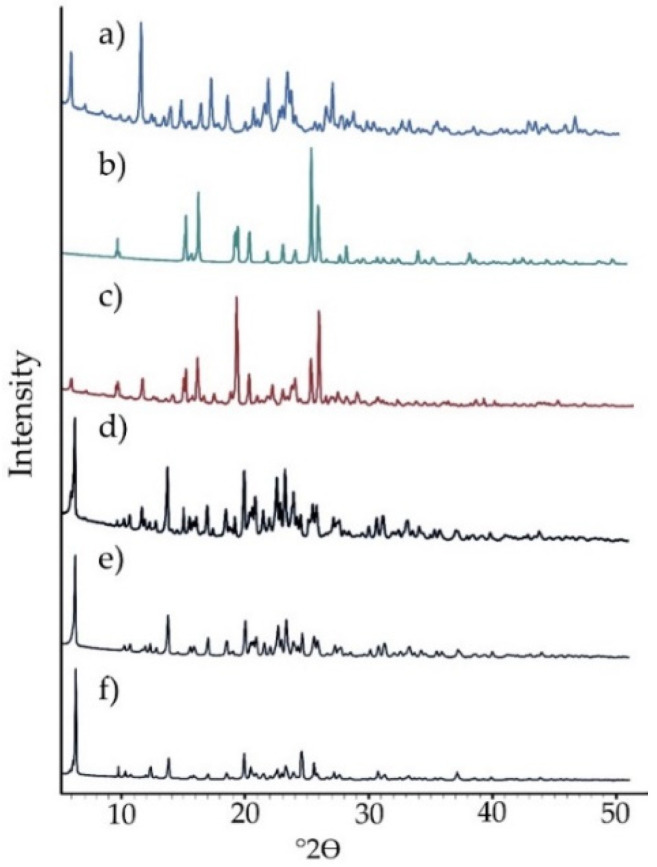
Powder diffractograms for (**a**) chlordiazepoxide, (**b**) saccharin, (**c**) chlordiazepoxide mixture with saccharin, and saccharin salts of chlordiazepoxide prepared using the following methods: (**d**) liquid-assisted grinding, (**e**) crystallization from ACN, and (**f**) crystallization from MeOH/ClMe.

As shown in Figure 2 and Table 1, the diffraction pattern of the mixture is the sum of the diffraction maxima for chlordiazepoxide and saccharin, which suggests that there was no interaction between both components after mixing. The diffraction pattern of the physical mixture displays both the signals characteristic of chlordiazepoxide at 2θ for the angles 5.79°, 11.50°, 22.00°, and 23.82° and saccharin at 2θ for the angles 9.50°, 15.00°, 19.06°, 20.10°, 25.08°, and 25.70°. In contrast, the salt obtained by grinding of the physical mixture with the addition of a small volume of ACN showed sharp, new diffraction peaks at 2θ for the angles 6.05°, 13.56°, 16.78°, 18.31°, 19.79°, 22.43°, 23.11°, 24.38°, and 25.35. Similarly, the saccharin salt of chlordiazepoxide crystallized from ACN was characterized by completely new signals at 2θ for the angles 6.09°, 13.56°, 16.80°, 18.30°, 19.80°, 22.44°, 23.13°, 24.40°, and 25.34°. Additionally, the saccharin salt of chlordiazepoxide crystallized from MeOH/ClMe was characterized only by new signals at 2θ for the angles 6.09°, 13.59°, 16.79°, 18.31°, 19.73°, 22.37°, 23.12°, 24.39°, and 25.38°. All salts showed one signal at 2θ for the angle approx. 20.70°, possibly from chlordiazepoxide.

Overall, intensive diffraction peaks characteristic of the physical mixture at 2θ for the angles 11.46, 15.90, 19.06 and 21.94 disappeared or reduced significantly in intensity in the saccharine salt of the chlordiazepoxide diffraction pattern. This implies that a new crystal phase was created.

**Figure 3:**
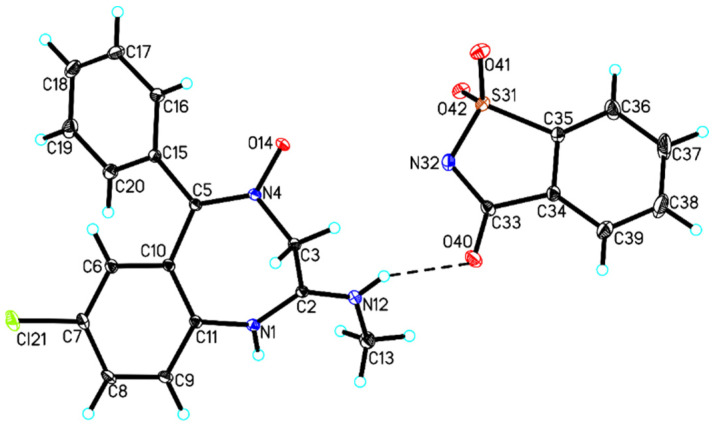
Molecular structure of saccharin salt of chlordiazepoxide showing the atom-labeling scheme (hydrogen bonds are represented by dashed lines).

Owing to measurements using the SCXRD system, it is possible to elaborate the structure of crystal salts on the basis of their diffraction images of the recorded reflection angles and the intensity of the deflected radiation beams. Thus, good-quality samples of saccharin salt of chlordiazepoxide monocrystals were selected for the SCXRD experiments. Selected crystallographic data for the examined salt are summarized in Table 2. These results showed that the saccharin salt of chlordiazepoxide is a crystalline substance and crystallizes in the orthorhombic *P*bca space group. The asymmetric part of the unit cell of the salt contains one chlordiazepoxide cation and one saccharin anion (Figure 3).

**Figure 4:**
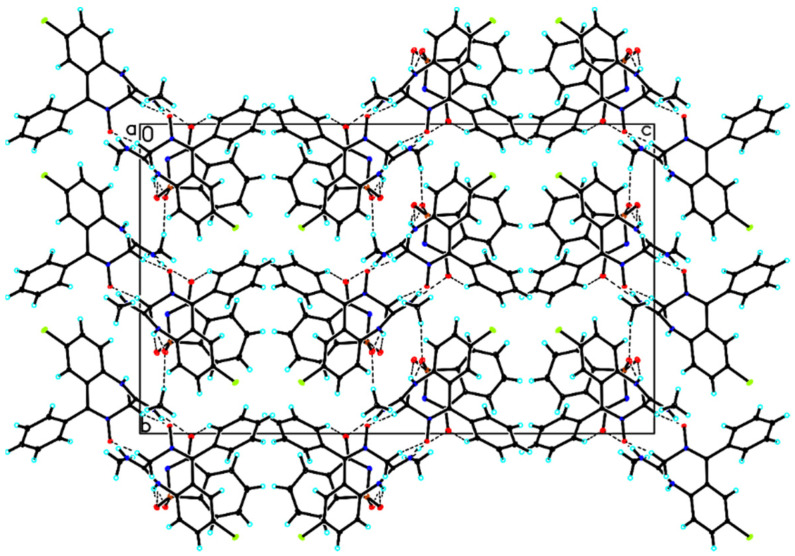
Crystal packing of saccharin salt of chlordiazepoxide viewed along the a-axis (hydrogen bonds are represented by dashed lines).

In the crystal of saccharin salt of chlordiazepoxide, an intramolecular C16–H16A···O14 hydrogen bond in chlordiazepoxide cation was observed, whereas chlordiazepoxide cation and saccharine anion interacted via strong N–H···O hydrogen bonds and weak C–H···O hydrogen bonds to produce blocks along the a-axis (Table 3, Figure 4). The neighboring blocks were linked via weak C_(chlordiazepoxide)_–H···π_(chlordiazepoxide)_ interaction to form a 3D framework (Table 4, Figure 4).

**Figure 5:**
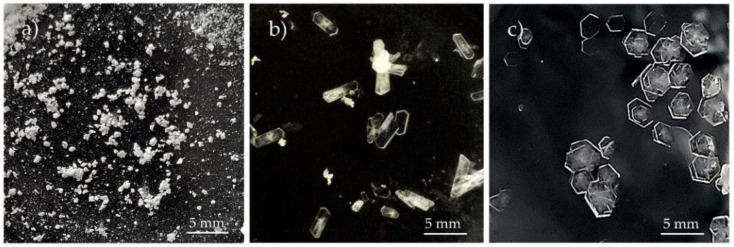
Images for saccharin salts of chlordiazepoxide prepared using the following methods: (**a**) liquid-assisted grinding, (**b**) crystallization from ACN, and (**c**) crystallization from MeOH/ClMe. Images were taken at 20 °C.

The appearance of the crystals of the saccharin salts of chlordiazepoxide is illustrated in Figure 5. These images suggest that the appearance of the crystals depends on the type of solvent used for the crystallization.

The fact of obtaining a new saccharin salt of chlordiazepoxide is also confirmed by the analysis of the state of knowledge described in the Cambridge Structural Database (CSD). CSD is a worldwide database containing a collection of all crystal structures of organic and organometallic compounds. Currently, the CSD database includes four crystal structures containing chlordiazepoxide: the structure of chlordiazepoxide [13], the structure of the chloride of chlordiazepoxide [26], the structure of the salt of chlordiazepoxide sulfate (VI) [27], and the solvate of chlordiazepoxide dichloromethane [28].

### 2.2. Thermoanalytical Measurements

**Figure 6:**
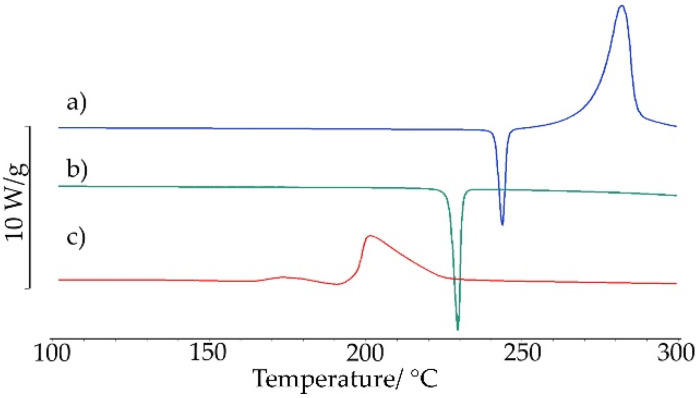
DSC curves for: (**a**) chlordiazepoxide, (**b**) saccharin, and (**c**) chlordiazepoxide mixture with saccharin. Measurements were taken at a heating rate of 10 °C/min in nitrogen.

**Figure 7:**
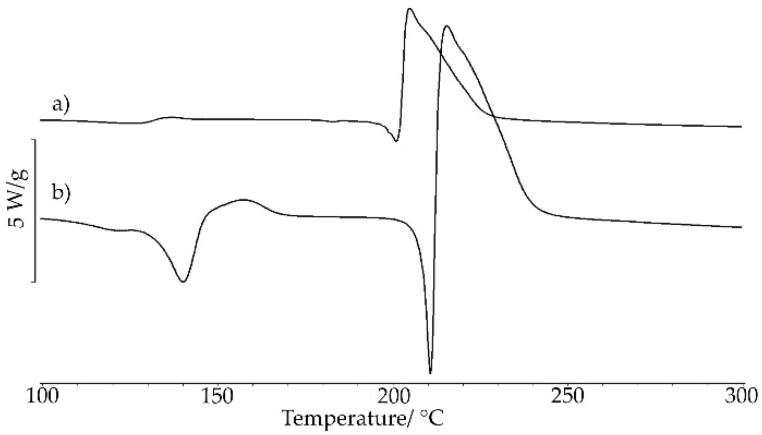
DSC curves for saccharin salt of chlordiazepoxide prepared by the liquid-assisted grinding method. Measurements were taken in nitrogen at a heating rate of (**a**) 10 °C/min and (**b**) 20 °C/min.

**Figure 8:**
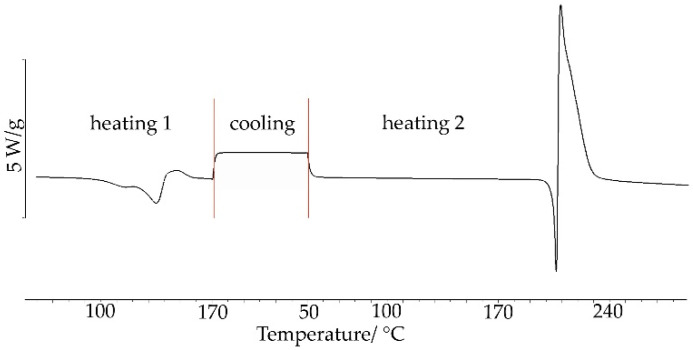
DSC curve for chlordiazepoxide mixture with saccharin prepared by the grinding with ACN for 30 min. Measurements were taken in nitrogen using heating, cooling, and re-heating programs.

**Figure 9:**
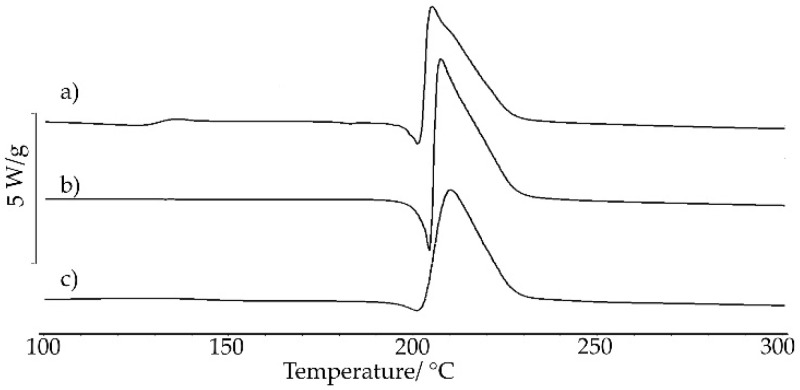
DSC curves for saccharin salts of chlordiazepoxide prepared using the following methods: (**a**) liquid-assisted grinding, (**b**) crystallization from ACN, and (**c**) crystallization from MeOH/ClMe. Measurements were taken at a heating rate of 10 °C/min in nitrogen.

Findings of DSC measurements of the examined samples are compiled in Table 5 and illustrated in Figure 6, Figure 7, Figure 8 and Figure 9. As shown in Figure 6a and Table 5, an endothermic DSC peak at *T_on_* = 240.8 °C reflects the melting of chlordiazepoxide. Then, the molten material undergoes thermal decomposition at *T_on_* = 274.6 °C, which confirms a sharp exothermic peak. In the case of saccharin (Figure 6b), a single endothermic peak at *T_on_* = 225.9 °C illustrates its melting. After melting, saccharin did not undergo any thermal changes over the temperature range studied. DSC measurements for the physical mixture of chlordiazepoxide with saccharin (Figure 6c) indicate that the DSC curve showed two weak signals: endothermic at *T_on_* = 154.0 °C probably due to eutectic formation and exothermic at *T_on_* = 165.1 °C assigned to salt crystallization. The newly formed saccharin salt of chlordiazepoxide melted at *T_on_* = 182.7 °C (endothermic peak) and then underwent thermal decomposition. Thermal degradation of salt characterized a large exothermic effect occurring in the temperature range of 200–230 °C (*T_on_* = 196.1 °C). The same sequence of thermal processes (endotherm, exotherm, endotherm) was also observed for the formation of co-crystals of hydroxybenzamides with benzoic acid derivatives [29] and chlordiazepoxide with *p*-aminobenzoic acid [30,31].

DSC curves for saccharin salt of chlordiazepoxide obtained by grinding with the addition of a small volume of ACN showed that the salt formation process was not complete. This is confirmed by two small DSC peaks (Figure 7a), an endothermic at *T_on_* = 118.9 °C and an exothermic at *T_on_* = 131.2 °C. These two effects may reflect a thermally induced process of salt formation. The endothermic effect indicates the formation of a eutectic mixture, from which the salt then crystallized (exothermic effect). Finally, the salt melted what confirms the endothermic effect at *T_on_* = 197.4 °C and then decomposed what reflects the exothermic peak at *T_on_* = 202.3 °C. This course of the DSC curve is also characteristic of a physical mixture whose components form a eutectic and then crystallize to form a salt. The eutectic mixture is probably made up of components that did not react completely when grinding with the addition of ACN. Repeated DSC measurements at a heating rate of 20 °C/min confirmed this course of salt formation (Figure 7b). Perhaps the process is more dynamic due to the high heating rate; therefore, there were two endothermic DSC peaks, at *T_on_* = 111.3 °C (very weak, its origin was not established) and at *T_on_* = 132.2 °C (eutectic formation). These peaks were followed by an exothermic effect at *T_on_* = 144.8 °C due to salt crystallization. The salt melted at *T_on_* = 207.5 °C (endothermic peak) and then decomposed at *T_on_* = 211.9 °C (exothermic peak).

The salt obtained by the grinding method was also explored using the heating, cooling, and re-heating measurements. As shown in Figure 8, during the first heating cycle, two small endothermic DSC peaks followed by an exothermic peak were recorded in the temperature range of 120–160 °C. The peaks can be attributed to the formation of a eutectic mixture (endothermic) from which the salt then crystallized (exothermic). No thermal effects were observed while cooling. On re-heating, sharp DSC peaks were recorded at higher temperatures than in the first heating: endothermic at *T_on_* = 203.04 °C due to salt melting and exothermic at *T_on_* = 206.15 °C associated with salt decomposition. This also implies that unreacted starting components as a result of grinding with ACN, in the first heating cycle, create a eutectic mixture from which salt crystallizes. The endothermic peak in the second heating cycle reflects the melting of the salt, formed both by grinding components with the addition of ACN and from unreacted components during the first heating of the sample. Then, the salt decomposes.

Figure 9 shows that on all DSC curves of saccharin salts of chlordiazepoxide obtained by different methods, a single DSC endothermic peak appeared due to the melting of the salt followed by an exothermic peak corresponding to the decomposition of the molten salt. As shown in Table 5, the salts melted in the temperature range from *T_on_* = 194.6 °C (salt crystallized from MeOH/ClMe) to *T_on_* = 201.2 °C (salt crystallized from ACN), i.e., in the range of 7 °C. Salt obtained by grinding with the addition of ACN melted at an intermediate temperature, i.e., at *T_on_* = 197.4 °C. The difference in melting points is due to different methods of salt preparation and the type of solvents used. This is consistent with literature data which report that the melting points may vary from a few to several degrees Celsius for measurements carried out with the same heating rate of the sample [31,32].

**Figure 10:**
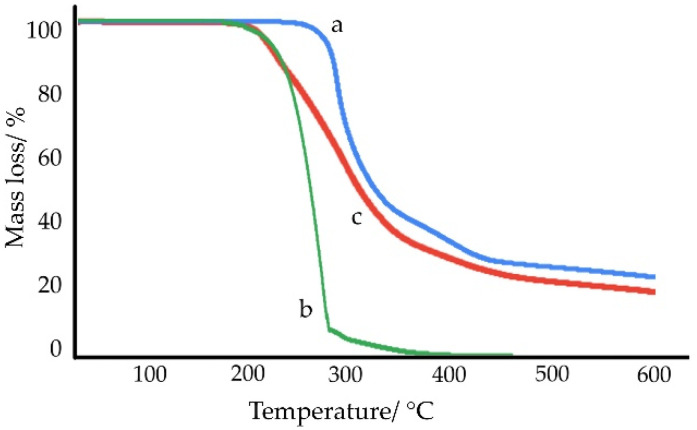
TGA curves for (**a**) chlordiazepoxide, (**b**) saccharin, and (**c**) chlordiazepoxide mixture with saccharin. Measurements were taken at a heating rate of 10 °C/min in nitrogen.

**Figure 11:**
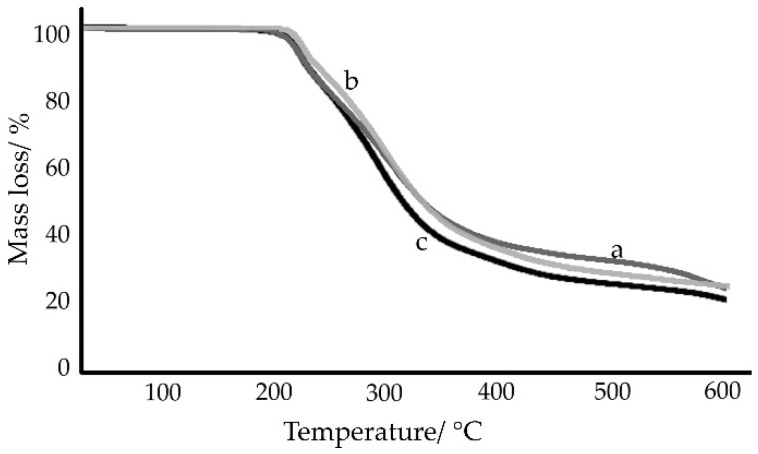
TGA curves for saccharin salts of chlordiazepoxide prepared using the following methods: (**a**) liquid-assisted grinding, (**b**) crystallization from ACN, and (**c**) crystallization from MeOH/ClMe. Measurements were taken at a heating rate of 10 °C/min in nitrogen.

Results of the TGA measurements of samples under study are shown in Figure 10 and Figure 11. When comparing the DSC and TGA findings, it should be taken into account that the DSC phase transition studies at temperatures up to 300 °C were performed in closed, flat-bottomed pans with perforated lids, while the TGA and open ceramic crucibles were used to study the thermal decomposition of samples (measurements up to 600 °C). The use of open crucibles facilitates the release of volatile substances or gaseous degradation products with increasing temperature. This means that in the case of an open crucible, mass losses are observed at a lower temperature than in the case of a closed pan. Therefore, the onset of mass loss of a sublimable substance may appear on the TGA curve at a temperature lower than the melting point of the same substance as determined by DSC.

TGA curves shown in Figure 10a revealed that chlordiazepoxide decomposed rapidly immediately after melting. The charred residue, approx. 24% at 600 °C, is probably to burn at higher temperatures. In the case of saccharin (Figure 10b), the mass loss began at 174.9 °C and at the melting point (*T_on_* = 225.9 °C), the mass loss was already approx. 10%. The obtained findings are consistent with the literature data. Carvalho et al. showed that the total mass loss of saccharin occurs in the temperature range from 152.8 to 372.3 °C, although saccharin only melts at 226.8 °C [33]. One-step mass loss of saccharin in the temperature range of 203–329 °C was also found by Ferreira et al. [34]. It is due to the sublimation of saccharin, and after melting at 221 °C, saccharin evaporates without thermal decomposition.

The TGA curve for the physical mixture of chlordiazepoxide and saccharin (Figure 10c) was very similar to the TGA curves for the saccharin salts of chlordiazepoxide (Figure 11) and clearly differed from the curves of the starting materials. This is in line with DSC experiments which show that when heated, chlordiazepoxide forms a eutectic with saccharin from which the salt crystallizes. The thermal decomposition of all salts showed at least two overlapping stages of mass loss. However, there was no clearly marked line between these successive stages. The first stage took place to approx. 250 °C with a mass loss between 12.3% (salt crystallized from ACN, Figure 11b) and 18.3% (salt obtained by grinding with ACN, Figure 11a). The second stage was accompanied by a mass loss of between 47.6% (salt obtained by grinding with ACN, Figure 11a) and 48.6% (salt crystallized from MeOH/ClMe, Figure 11c) and took place to approx. 380 °C for salts crystallized from solvents and up to 460 °C for salt obtained by grinding with ACN. Overall, TGA experiments showed that salt can be obtained both by heating a physical mixture and by grinding or crystallizing starting materials.

### 2.3. Infrared Spectroscopic Measurements

**Figure 12:**
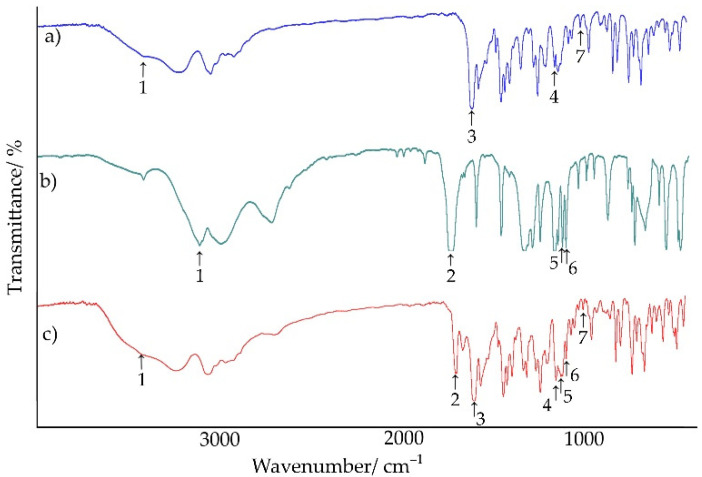
FT-IR spectra for (**a**) chlordiazepoxide, (**b**) saccharin, and (**c**) chlordiazepoxide mixture with saccharin.

**Figure 13:**
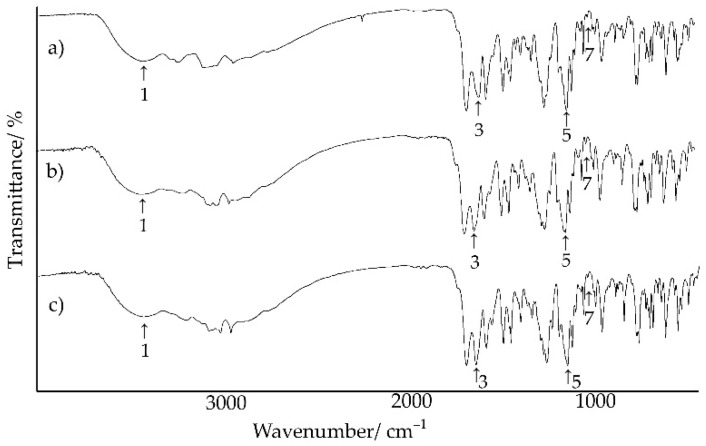
FT-IR spectra for saccharin salts of chlordiazepoxide prepared using the following methods: (**a**) liquid-assisted grinding, (**b**) crystallization from ACN, and (**c**) crystallization from MeOH/ClMe.

FT-IR spectra of chlordiazepoxide, saccharin, their physical mixture, and salts obtained by different methods are presented in Figure 12 and Figure 13, while the results of their interpretation are compiled in Table 6. It summarizes characteristic group frequencies, mainly of functional groups capable of forming hydrogen bonds. In the case of chlordiazepoxide, absorption bands were distinguished at 3426.4, 1624.8, 1170.5, and 1029.4 cm^−1^ [31]. Saccharin was characterized by absorption bands at 2094.1, 1716.9, 1139.9, and 1121.4 cm^−1^ [32]. Moreover, the position of the absorption bands in the spectrum of the physical mixture largely coincided with the position of the characteristic bands of chlordiazepoxide and saccharin (Figure 12). However, the disappearance of the saccharin band at 3094.1 cm^−1^ and the shift of the band at 1139.9 cm^−1^ towards higher wavenumber values, i.e., 1141.6 cm^−1^, was observed. Overall, the FTIR spectrum of the physical mixture showed that gentle mixing of starting components at room temperature did not result in the formation of chemical bonds or physical interactions between both materials, and thus, no saccharin salt of chlordiazepoxide was formed under these conditions. Only heating the mixture of starting components during DSC analysis resulted in the formation of the salt.

As shown in Figure 13, regardless of the method of obtaining the saccharin salt of chlordiazepoxide, their FT-IR spectra coincided. However, they differed significantly from the spectra of the starting substances and physical mixture (Figure 12). The characteristic absorption bands of saccharin at 3094.1 cm^−1^ (–NH), 1716.9 cm^−1^ (C=O), and 1121.4 cm^−1^ (–SO_2_) and of chlordiazepoxide at 1170.5 cm^−1^ (C–N) disappeared. The band associated with the C=N stretching vibration of the chlordiazepoxide ring shifted towards a higher wavenumber. Moreover, the presence of unreacted starting components in the salt obtained by grinding with the addition of ACN (DSC results) was not confirmed in the FT-IR spectrum of this salt (Figure 13a). This spectrum did not differ from the FT-IR spectra of the salts obtained by crystallization from ACN or from MeOH/ClMe. Only insignificant differences were found in the shape and intensity of some bands in the range of 3300–2800 cm^−1^.

**Figure 14:**
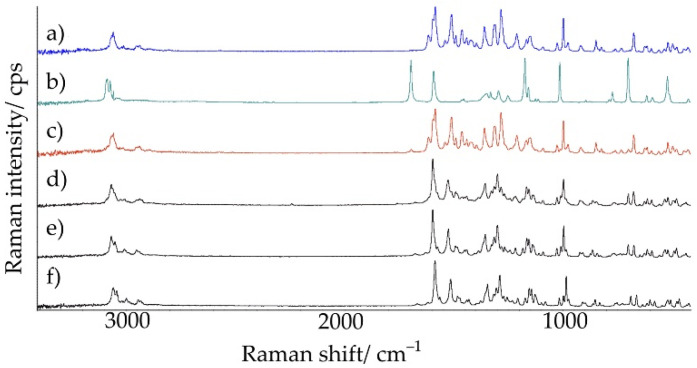
Raman spectra for (**a**) chlordiazepoxide, (**b**) saccharin, (**c**) chlordiazepoxide mixture with saccharin, and saccharin salts of chlordiazepoxide prepared using the following methods: (**d**) liquid-assisted grinding, (**e**) crystallization from ACN, and (**f**) crystallization from MeOH/ClMe.

Raman spectra of the examined samples are shown in Figure 14, while the results of their interpretation are listed in Table 7. It shows the characteristic shifts of the Raman bands of chlordiazepoxide and saccharin, their physical mixture, and salts obtained by various methods. In the case of chlordiazepoxide, Raman bands could be distinguished at 3064.6, 1621.4, and 1589.8 cm^−1^ [31,35]. Saccharin was characterized by bands at 1700.7, 1596.7, and 1178.7 cm^−1^ [36]. The data presented in Table 7 differ slightly from the literature data in the range of up to 10 cm^−1^. The Raman spectrum of the physical mixture showed all characteristic bands of chlordiazepoxide and a weak but characteristic signal of saccharin at 1696.6 cm^−1^. On the other hand, the spectra of the salts clearly differed from the spectra of the starting components and physical mixture. The bands that were present in the Raman spectra of the salts are the vibrations stretching the –CH groups of chlordiazepoxide and the vibrations stretching the C–C bonds of saccharin. The shifting of the bands depends on the method of salt preparation and the type of solvent. Salt obtained by grinding with the addition of ACN and salt crystallized from ACN showed bands at 3073.0 and 1600.8 cm^−1^, and at 3073.9 and 1601.2 cm^−1^, respectively. In turn, salt crystallized from MeOH/ClMe showed these bands at lower wavenumbers, i.e., at 3063.6 and 1591.1 cm^−1^. In the whole studied spectral range, the shape of some bands changed, especially in the range of 1700–1100 cm^−1^.

FT-IR and Raman explorations confirmed the possibility of obtaining saccharin salt of chlordiazepoxide. The FT-IR and Raman spectra of the physical mixture consisted of bands of the starting components, while the spectra of the salts implied the presence of hydrogen bonds between the methylamino group and the nitrogen of the aromatic chlordiazepoxide ring, and the oxygen of the carbonyl group and one of the oxygens of the sulfone group of saccharin. Moreover, the complete disappearance of the N–H band in the FT-IR spectrum of saccharin may indicate a transfer of this proton towards chlordiazepoxide. On the other hand, the disappearance of the aromatic bond band in the chlordiazepoxide ring in the Raman spectrum may suggest the formation of hydrogen bonds between successive salt molecules in space (intermolecular hydrogen bonds).

### 2.4. Preliminary Solubility Assessment

The water solubility of the saccharin salt of chlordiazepoxide was not assessed using spectrophotometric and chromatographic procedures due to the negligible solubility of chlordiazepoxide in water. For this reason, the gravimetric method was used and the results obtained are summarized in Table 8.

These findings showed a significant improvement in the water solubility of chlordiazepoxide as a saccharin salt. However, the solubility insignificantly depended on the method of the salt preparation. The salts prepared with ACN showed better solubility. The average solubility of the saccharin salts of chlordiazepoxide was 7.29 mg/mL. In addition, the data obtained indicate that, compared with the water solubility of chlordiazepoxide, the solubility of the drug substance as a salt increased approx. 50-fold. Such a large increase in the solubility of the drug substance in the form of an organic salt is consistent with the literature data [11,18]. For example, Bhatt et al. showed that the water solubility of quinine as a salt with saccharin increased 54-fold, while that of haloperidol increased over 600-fold [11].

## 3. Materials and Methods

### 3.1. Materials

Chlordiazepoxide was kindly supplied by Polfa Tarchomin (Warsaw, Poland). Saccharin was received from Acros Organics (Geel, Belgium). Since the purity of materials was above 99%, both compounds were used without further purification. Methanol, ethylene chloride, and acetonitrile (pure for analysis) were bought from POCH (Gliwice, Poland).

#### 3.1.1. Sample Preparation

A binary physical mixture of chlordiazepoxide and saccharin was prepared at a 1:1 molar ratio. Both components were accurately weighed using Mettler Toledo XA105 Dual Range balance (Schwerzenbach, Switzerland), then transferred to microtubes (Sarstedt, Nümbrecht, Germany) and mixed using a laboratory stirrer (Kamush, Gdansk, Poland) for 15 min at 20 rpm. Chlordiazepoxide salt with saccharin was prepared using a physical mixture as starting material in two ways, i.e., the method of grinding with a small volume of solvent and the method of crystallization with complete evaporation of a solvent.

The saccharin salt of chlordiazepoxide obtained by grinding with the addition of a small volume of solvent was prepared as follows: 150 mg of the physical mixture was placed in a microtube, two agate beads with a diameter of 5 mm (Eqiumed, Krakow, Poland) were added, and a small volume (3 drops) of acetonitrile (ACN) was added. The mixture was ground with a laboratory stirrer for 30 min at 20 rpm. Then, the microtube was left open at room temperature in order to evaporate the solvent (about 24 h).

To prepare saccharin salt of chlordiazepoxide by crystallization with complete evaporation of a solvent, two variants of the method were applied. In the former case, for a beaker with a capacity of 10 mL, 200 mg of the physical mixture and 2 mL of ACN were added. The content of the beaker was stirred and heated to the boiling point of a solvent at 82 °C and then left at room temperature for several days for the salt to crystallize and the ACN to be completely evaporated. After this time, colorless crystals of the saccharin salt of chlordiazepoxide were collected. In the latter case, 600 mg of the physical mixture was placed in a beaker with a capacity of 100 mL and dissolved in 30 mL of a mixture of methanol (MeOH) with methylene chloride (ClMe) (5:1, v:v). The beaker was covered with foil, leaving a small outlet, and then placed in the refrigerator for 5 days. After this time, large, hexagonal, colorless crystals appeared. The beaker with the crystals was left at room temperature for 5 days for complete evaporation of the solvent.

#### 3.1.2. Solubility Assessment

Water solubility of the examined samples was assessed by a gravimetric method [37]. First, 10-mL aliquots of saturated solutions of the compounds under study (chlordiazepoxide, saccharin and salts) were mixed with a magnetic stirrer (UniStirrer 3, LLG Labware, Meckenheim, Germany) for 24 h at room temperature. The solutions were then filtered (filter paper, 60 g/m^2^, Ahlstrom Munksjö, Bärenstein, Germany), and 2 mL of the filtrate was transferred to a weighing vessel and left at room temperature until the solvent was completely evaporated (about 7 days). From the difference in the weight of the empty weighing vessel and after evaporation of the water, the weight of the dissolved substance in 2 mL of the solvent was calculated.

### 3.2. Methods

#### 3.2.1. Powder X-ray Diffraction (PXRD)

PXRD patterns of the powdered samples under study (chlordiazepoxide, saccharin, their physical mixture and salts) were collected using the Philips X’Pert PRO MPD system (Almelo, The Netherlands) with Cu-Kα radiation at a wavelength of 1.541 Å. Instrumental parameters were as follows: 2θ angle range of 7–55°, counting time 3 s per step, counting step (2θ) 0.02°, X-ray tube setting at 40 kV, and 30 mA. The diffractometer was calibrated using the polycrystalline silicon standard.

#### 3.2.2. Single Crystal X-ray Diffraction (SCXRD)

Diffraction data for single crystals of saccharin salts of chlordiazepoxide were collected on an X-ray Diffractometer Gemini R Ultra (Oxford Diffraction, Yarnton, UK) equipped with a Ruby CCD detector. Measurements were carried out at *T* = 295(2) K using MoKα (λ = 0.71073 Å) as a radiation source. The lattice parameters were obtained using CrysAlis CCD [38], whereas data were reduced using CrysAlis RED software [38]. The structure was solved and refined using the SHELX programs [39].

H-atoms bound to N-atoms were located on a difference Fourier map and refined freely. H-atoms bound to aromatic C-atoms were placed geometrically and refined using a riding model with C–H = 0.93/0.96 Å and U_iso_(H) = 1.2U_eq_(C) (C–H = 0.96 Å and U_iso_(H) = 1.5U_eq_(C) for the methyl group). All interactions were calculated using the PLATON program (ver. 181115) [40], whereas to prepare the molecular graphics, the ORTEPII [41], PLUTO-78 [42], and Mercury (ver. 2020.2.0) [43] programs were used.

Full crystallographic details for the title compound (saccharin salt of chlordiazepoxide) were deposited in the Cambridge Crystallographic Data Center (deposition No. CCDC 2205773) and they may be obtained from www: http://www.ccdc.cam.ac.uk (accessed on 12 September 2022), e-mail: deposit@ccdc.cam.ac.uk or The Director, CCDC, 12 Union Road, Cambridge, CB2 1EZ, UK.

Images of the crystals were taken with an Olympus BX41 polarizing microscope and an SC30 color digital video camera coupled with Olympus CellA software (Olympus, Shinjuku, Japan). Pictures were taken at room temperature.

#### 3.2.3. Differential Scanning Calorimetry (DSC)

DSC curves of the chlordiazepoxide, saccharin, their physical mixture, and salts prepared by different methods were obtained using a heat-flux 822e Mettler Toledo DSC calorimeter (Schwerzenbach, Switzerland) coupled with STARe software ver. 15.00 (Mettler Toledo, Schwerzenbach, Switzerland). All the samples in similar amounts (4–5 mg) were weighed and closed in flat-bottomed pans with perforated lids. Measurements were run at a 10 °C/min heating rate over the temperature range of 50–300 °C, in nitrogen at a flux rate of 70 mL/min. Using heating, cooling, and re-heating measurements, the samples were heated in the temperature range 50–170 °C at a rate of 20 °C/min, kept at 170 °C for 2 min, then cooled to 50 °C at a rate of 20 °C/min, held at 50 °C for 2 min, then re-heated to 300 °C at a rate of 20 °C/min. The calorimeter was calibrated using indium and zinc reference materials.

#### 3.2.4. Thermogravimetric Analysis (TGA)

TGA curves of the samples examined were obtained with the use of a TGA 8000 Perkin Elmer device (Waltam, MA, USA) coupled with PYRIS software ver. 13.4.0 (Perkin Elmer, Waltam, MA, USA). About 5–6 mg of samples was weighed and placed in open ceramic crucibles. The samples were analyzed over the temperature range from 25 to 600 °C at a heating rate of 10 °C/min under nitrogen. The flow rate of the carrier gas was 10 mL/min.

#### 3.2.5. Fourier Transform Infrared (FT-IR)

An FT-IR spectrometer Nicolet 380 Thermo Fischer Scientific (Madison, WI, USA) coupled with a DTGS KBr detector and OMNIC 7 software was used to record the FT-IR spectra of the examined samples. For the preparation of tablets containing 1 mg of sample and 100 mg of KBr (Merck, Darmstadt, Germany), a hydraulic press was used (Specac, Orpington, UK). FT-IR spectra were recorded in the spectral range of 4000–400 cm^−1^ with a resolution of 4 cm^−1^ at room temperature. The background spectrum was checked before each measurement.

#### 3.2.6. Raman Spectroscopy

A Thermo Fisher Scientific DXR Smart Raman spectrometer (Madison, WI, USA) coupled with OMNIC software (Thermo Fisher Scientific, Madison, WI, USA) was used to record the Raman spectra of the samples under study. The spectrometer was equipped with a 15 mW DXR 780 nm laser with 25 µm aperture width, a CCD detector (Thermo Fisher Scientific, Madison, WI, USA), and a Raleigh filter (Thermo Fisher Scientific, Madison, WI, USA). Raman spectra were acquired in the range of 3413–99 cm^−1^ with a resolution of 2 cm^−1^. The exposure time was 1 s (twice).

## 4. Conclusions

On the basis of experiments carried out using X-ray diffractometric, thermoanalytical, and spectroscopic techniques, it was proved that chlordiazepoxide forms a salt with saccharin in a molar ratio of 1:1. The crystalline salt can be obtained by various methods: grinding with the addition of acetonitrile and crystallization from acetonitrile or a mixture of methanol with methylene chloride. Salt may also be formed upon heating a physical mixture of both starting compounds under DSC conditions. The saccharin salt crystallized in the orthorhombic *P*bca space group and was characterized by containing a chlordiazepoxide cation and a saccharin anion. The chlordiazepoxide cation and the saccharin anion interacted through strong N–H···O hydrogen bonds and weak C–H···O hydrogen bonds. On the other hand, the disappearance of the N–H band in the FT-IR spectrum of saccharin may indicate a shift of this proton towards chlordiazepoxide. The melting point of the salts differed from that of the starting compounds. The slight difference in the melting point of the salt is due to the different methods of salt preparation and the type of solvents used. Thermal decomposition of the salt began above 200 °C and showed at least two overlapping stages of mass loss.

## 5. Patents

Title compound is protected by Polish patent application no. P.440394, submitted by the authors of this paper.

## Figures and Tables

**Table 1:** Selected powder X-ray crystallographic data for chlordiazepoxide, saccharin, chlordiazepoxide mixture with saccharin, and saccharin salts of chlordiazepoxide prepared by various methods.

Samples	Diffraction Angle 2θ (°)	d-Spacing (Å)	Relative Intensity (%)
Chlordiazepoxide	5.75, 11.49, 13.91, 14.80, 16.41, 17.26, 20.72, 21.95, 23.52, 27.20	15.36, 7.70, 6.36, 5.98, 5.40, 5.13, 4.28, 4.05, 3.78, 3.28	49.27, 100.00, 18.87, 27.06, 25.68, 49.94, 22.45, 52.74, 56.73, 51.75
Saccharin	9.49, 14.99, 16.03, 19.07, 20.13, 25.13, 25.70	9.31, 5.91, 5.53, 4.65, 4.41, 3.54, 3.46	10.35, 31.96, 61.01, 23.50, 26.27, 100.00, 47.32
Binary physical mixture	5.69, 9.46, 11.46, 14.93, 15.90, 19.06, 20.05, 21.94, 23.76, 25.04, 25.68	15.51, 9.34, 7.72, 5.93, 5.57, 4.65, 4.43, 4.05, 3.74, 3.55, 3.47	12.25, 14.66, 21.05, 24.01, 38.79, 100.00, 29.75, 17.33, 23.71, 49.09, 90.51
Salt prepared by liquid-assisted grinding	6.05, 13.56, 16.78, 18.31, 19.79, 20.70, 22.43, 23.11, 24.38, 25.35	14.60, 6.52, 5.27, 4.84, 4.48, 4.28, 3.96, 3.84, 3.65, 3.51	100.00, 76.46, 34.68, 30.36, 77.96, 41.42, 56.35, 76.20, 22.94, 36.77
Salt prepared by crystallization from ACN	6.09, 13.56, 16.80, 18.30, 19.80, 20.71, 22.44, 23.13, 24.40, 25.34	14.50, 6.52,5.27, 4.84, 4.48, 4.29, 3.96, 3.84, 3.64, 3.51	100.00, 47.70, 20.68, 18.81, 44.31, 22.46, 38.56, 46.19, 28.87, 24.01
Salt prepared by crystallization from MeOH/ClMe	6.09,13.59, 16.79, 18.31, 19.73, 20.66, 22.37, 23.12, 24.39, 25.38	14.49, 6.51, 5.28, 4.84, 4.50, 4.30, 3.97, 3.84, 3.65, 3.51	100.00, 17.74, 4.70, 5.53, 26.29, 4.96, 9.06, 12.35, 29.34, 17.01

**Table 2:** Crystal data and structure refinement for saccharin salt of chlordiazepoxide.

Crystal Data	Saccharin Salt of Chlordiazepoxide
Chemical formula	C_16_H_15_ClN_3_O·C_7_H_4_NSO_3_
Formula weight/g·mol^−1^	482.93
Crystal system	orthorhombic
Space group	Pbca
a/Å	8.9526(2)
b/Å	17.5658(4)
c/Å	29.1681(6)
α/°	90
β/°	90
γ/°	90
V/Å^3^	4586.98(17)
Z	8
T/K	295(2)
λMo/Å	0.71073
ρ_calc_/g·cm^−3^	1.399
F(000)	2000
µ/mm^−1^	0.297
θ range/°	3.30–25.00
Completeness θ/%	99.7
Reflections collected	32231
Reflections unique	4036 [R_int_ = 0.0305]
Data/restraints/parameters	4036/0/305
Goodness of fit on F^2^	1.119
Final R_1_ value (I > 2σ(I))	0.0429
Final wR_2_ value (I > 2σ(I))	0.0947
Final R_1_ value (all data)	0.0477
Final wR_2_ value (all data)	0.0973
CCDC number	2205773

**Table 3:** Hydrogen bond geometry for saccharin salt of chlordiazepoxide.

D–H···A	d(D–H) (Å)	d(H···A) (Å)	d(D···A) (Å)	∠D–H⋯A (°)
N1–H1···O42^i^	0.82(2)	2.10(3)	2.869(2)	157(2)
N12–H12···O40	0.82(2)	1.99(2)	2.734(3)	151(2)
C3–H3A···O41^ii^	0.97	2.39	3.340(3)	166
C3–H3B···O14^ii^	0.97	2.36	3.274(3)	156
C13–H13B···N32^i^	0.96	2.62	3.518(3)	155
C13–H13C···O41^iii^	0.96	2.40	3.219(3)	142
C16–H16A···O14*	0.93	2.57	2.924(3)	103
C16–H16A···O40^ii^	0.93	2.53	3.359(3)	149

Symmetry code: (i) 2-x, 1-y, 1-z; (ii) 1-x, 1-y, 1-z; (iii) 3/2-x, -1/2+y,z.

**Table 4:** C–X···π interaction geometry for saccharin salt of chlordiazepoxide.

C–X···Cg	d(X···Cg) (Å)	d(C···Cg) (Å)	∠C–X···Cg (°)
C8–H8A···Cg1^iii^	3.17	3.970(3)	145

Symmetry code: (iii) 3/2-x, -1/2+y,z. Cg denote the C15-C20 ring centroid.

**Table 5:** Characteristic temperatures and heats of transitions acquired from the DSC peaks for chlordiazepoxide, saccharin, chlordiazepoxide mixture with saccharin, and saccharin salts of chlordiazepoxide prepared by different methods. Measurements were taken at a heating rate of 10 °C/min in nitrogen.

Samples	*T_on_* (°C)	*T_p_* (°C)	Δ*H* (J/g)
Chlordiazepoxide	240.8	241.8	103.6 endo
	274.6	284.2	435.7 exo
Saccharin	225.9	226.8	156.4 endo
Binary physical mixture	154.0	158.9	8.5 endo
	165.1	172.8	15.8 exo
	182.7	189.7	11.4 endo
	196.1	201.4	300.6 exo
Salt prepared by liquid-assisted grinding	118.9	126.8	3.7 endo
	131.2	135.8	3.4 exo
	197.4	200.8	17.4 endo
	202.3	205.6	295.6 exo
Salt prepared by crystallization from ACN	201.2	203.7	30.3 endo
	204.9	208.1	324.1 exo
Salt prepared by crystallization from MeOH/ClMe	194.6	200.9	12.3 endo
	203.3	210.6	309.7 exo

*T_on_*—the onset temperature; *T_p_*—the peak temperature; Δ*H*—the heat of transition.

**Table 6:** Characteristic absorption bands acquired from the FT-IR spectra for chlordiazepoxide, saccharin, chlordiazepoxide mixture with saccharin, and saccharin salts of chlordiazepoxide prepared by different methods.

Assignment of Absorption Bands	Band Numbers	Samples					
		Chlordiazepoxide	Saccharin	Binary Physical Mixture	Salt Prepared by Liquid- Assisted Grinding	Salt Crystallized from ACN	Salt Crystallized from MeOH/ ClMe
–NH stretching	1	3426.4	3094.1	3427.5	3427.4	3428.0	3427.6
C=O stretching	2	–	1716.9	1719.4	–	–	–
C=N stretching of ring	3	1624.8	–	1624.7	1616.5	1635.1	1635.3
C–N symmetric	4	1170.5	–	1177.3	–	–	–
–SO_2_ asymmetric stretching	5	–	1139.9	1141.6	1142.0	1139.7	1139.2
–SO_2_ symmetric stretching	6	–	1121.4	1121.7	–	–	–
C–N asymmetric stretching	7	1029.4	–	1029.8	1030.9	1030.4	1031.5

**Table 7:** Characteristic Raman shifts for chlordiazepoxide, saccharin, chlordiazepoxide mixture with saccharin, and saccharin salts of chlordiazepoxide prepared by various methods.

Assignment of Absorption Bands	Samples					
	Chlordiazepoxide	Saccharin	Binary Physical Mixture	Salt Prepared by Liquid- Assisted Grinding	Salt Crystallized from ACN	Salt Crystallized from MeOH/ ClMe
–CH stretching	3064.6	–	3064.2	3073.0	3073.9	3063.6
C=O stretching	–	1700.7	1696.6	–	–	–
C=N stretching	1621.4	–	1620.7	–	–	–
C–C stretching	–	1596.7	–	1600.8	1601.2	1591.1
aromatic ring	1589.8	–	1589.9	–	–	–
–SO_2_ stretching	–	1178.7	–	–	–	–

**Table 8:** Water solubility of chlordiazepoxide, saccharin, and saccharin salts of chlordiazepoxide prepared by different ways.

Samples	Solubility in Water (mg/mL ± SD)
Chlordiazepoxide	0.12 ± 0.02
Saccharin	2.93 ± 0.04
Salt prepared by liquid-assisted grinding	7.70 ± 0.01
Salt prepared by crystallization from ACN	7.52 ± 0.22
Salt prepared by crystallization from MeOH/ClMe	6.65 ± 0.48

## Data Availability

Data is contained within this article.
